# A phase 1 trial of nebulised heparin in acute lung injury

**DOI:** 10.1186/cc6894

**Published:** 2008-05-06

**Authors:** Barry Dixon, John D Santamaria, Duncan J Campbell

**Affiliations:** 1 Department of Intensive Care, St Vincent's Hospital, 41 Victoria Parade, Melbourne 3065, Australia; 2 StVincent's Institute of Medical Research, 41 Victoria Parade, Melbourne 3065, Australia

## Abstract

**Introduction:**

Animal studies of acute lung injury (ALI) suggest nebulised heparin may limit damage from fibrin deposition in the alveolar space and microcirculation. No human studies have been undertaken to date. We assessed the feasibility, safety and potential anticoagulant effects of administration of nebulised heparin to patients with ALI.

**Methods:**

An open label phase 1 trial of four escalating doses of nebulised heparin was performed. A total of 16 ventilated patients with ALI were studied. The first group was administered a total of 50,000 U/day, the second group 100,000 U/day, the third group 200,000 U/day and the fourth group 400,000 U/day. Assessments of lung function included the PaO_2_/FiO_2 _ratio, lung compliance and the alveolar dead space fraction. Monitoring of anticoagulation included the activated partial thromboplastin time (APTT) and the thrombin clotting time. Bronchoalveolar lavage fluid was collected and the prothrombin fragment and tissue plasminogen activator levels were assessed. Analysis of variance was used to compare the effects of dose.

**Results:**

No serious adverse events occurred for any dose. The changes over time for the PaO_2_/FiO_2 _ratio, lung compliance and the alveolar dead space fraction levels were similar for all doses. A trend to increased APTT and thrombin clotting time levels was present with higher doses (*P *= 0.09 and *P *= 0.1, respectively). For the highest dose, the APTT reached 64 seconds; following cessation of nebulised heparin, the APTT fell to 39 seconds (*P *= 0.06). In bronchoalveolar lavage samples a trend to reduced prothrombin fragment levels was present with higher doses (*P *= 0.1), while tissue plasminogen activator levels were similar for all doses.

**Conclusion:**

Administration of nebulised heparin to mechanically ventilated patients with ALI is feasible. Nebulised heparin was not associated with any serious adverse events, and at higher doses it increased APTT levels. Larger trials are required to further investigate the safety and efficacy of nebulised heparin. In these trials due consideration must be given to systemic anticoagulant effects.

**Trial registration:**

Australian Clinical trials registry ACTRN12606000388516.

## Introduction

Acute lung injury (ALI) is a serious clinical problem. Estimates are that 190,600 cases of ALI develop in the United States each year, which are associated with 74,500 deaths and 3.6 million hospital days [1]. The 28-day mortality for ALI is 32% [2]. There is currently no method to prevent or treat ALI

ALI is characterised by the rapid onset of respiratory distress in the setting of an inflammatory insult to the lungs [3,4]. Inflammatory insults include sepsis, trauma, hypotension, cardiopulmonary bypass, pancreatitis, aspiration and multiple transfusions. Septic insults are by the commonest cause of ALI. Pneumonia triggers 30% of cases, and sepsis elsewhere in the body causes 32% of cases [2]

One mechanism by which inflammation causes ALI is deposition of fibrin in the alveolar space and microcirculation. Fibrin deposition in the alveolar sacs gives rise to a hyaline membrane, and deposition in the microvasculature results in thrombosis [5-14]. Nebulised heparin may limit fibrin deposition in the alveolar space and microcirculation through heparin's anticoagulant and fibrinolytic actions [15-18]. Studies in animal models of ALI have demonstrated that nebulised heparin improved the PaO_2_/FiO_2 _ratio and reduced histological damage [19,20]. In addition, in the setting of lung injury triggered by cardiac surgery, a preoperative heparin infusion reduced evidence of pulmonary microvascular thrombosis [21].

We are unaware of previous trials of nebulised heparin in patients with ALI. We therefore undertook the present trial to assess the feasibility, safety and potential anticoagulant effects of nebulised heparin in mechanically ventilated patients with ALI. In addition, we assessed the effects on intrapulmonary coagulation activation and fibrinolysis.

## Materials and methods

The study was approved by the St Vincent's Hospital Human Research Ethics Committee. Consent was obtained from the patient or next of kin before participation in the study.

The present study was an open-label, escalating-dosage phase 1 trial of nebulised heparin (heparin sodium, 25,000 U/ml; CP Pharmaceuticals Ltd, Wrexham, UK) in mechanically ventilated patients with ALI. Four doses were studied. Each dose was assessed in four patients over 2 days. The first group was administered 50,000 U/day, as 25,000 U 12 hourly (four nebulisations); the second group received 100,000 U/day, as 50,000 U 12 hourly (four nebulisations); the third group received 200,000 U/day, as 100,000 U 12 hourly (four nebulisations); and the fourth group was administered 400,000 U/day, as 100,000 U 6 hourly (eight nebulisations). The final nebulisation of heparin was administered at 36 hours from baseline in the 50,000 U/day, 100,000 U/day and 200,000 U/day groups, and at 42 hours in the 400,000 U/day group.

### Subjects

We studied patients admitted to the intensive care unit that met the following inclusion and exclusion criteria.

The inclusion criterion was the initiation of mechanical ventilation for acute respiratory dysfunction characterised by a PaO_2_/FiO_2 _ratio < 300 mmHg, where the acute respiratory dysfunction was primarily due to a direct or indirect inflammatory insult to the lung.

The exclusion criteria were > 48 hours since the inclusion criterion was met; hypoxemia predominantly due to a cause other than ALI, such as congestive heart failure, pulmonary embolism, chronic obstructive airways disease or asthma; systemic anticoagulation (including activated protein C), potential need for haemofiltration and therefore anticoagulation; pulmonary haemorrhage in the previous 3 months, uncontrolled bleeding, significant bleeding disorder; allergy to heparin, including heparin-induced thrombocytopenia; age < 18 years or > 85 years; patient unlikely to survive 96 hours; bronchoscopy not possible due to severe hypoxia; previous intubation and ventilation during current admission; noninvasive ventilation for more than 36 hours prior to intubation; or pregnancy.

### Nebulisation

Heparin was nebulised with an Aeroneb Pro Nebulizer (Aerogen Ltd, Galway, Ireland) over 30 minutes. The nebuliser was placed in the inspiratory line 12 cm from the Y of the circuit. The heat and moisture exchanger was removed during nebulisation. Patients were ventilated in a pressure-support mode of ventilation and upper pressure levels were maintained at or below 35 cmH_2_O.

### Lung function

The P_a_O_2_/F_i_O_2 _ratio, lung compliance and the alveolar dead space fraction were measured at baseline and at 2, 6, 10, 14, 18, 22, 26, 30, 34, 28, 42 and 46 hours. We measured the alveolar dead space fraction because previous studies have suggested this variable may reflect the extent of microvascular thrombosis in ALI [21,22]. Evidence of blood staining of respiratory secretions was assessed by the bedside nurse following routine pulmonary suctioning.

### Anticoagulant effects

The activated partial thromboplastin time (APTT) and the thrombin clotting time (TCT) were assessed at the same time points as those of lung function, and at 50, 54 and 58 hours in the 400,000 U/day group.

### Lung haemostatic responses

Prothrombin fragments (PTF) and tissue plasminogen activator (t-PA) levels in bronchoalveolar lavage (BAL) fluid were assessed at baseline and following the final nebulisation (BAL was undertaken on average 7.6 ± 5 hours following the final nebulisation).

### Lung compliance and alveolar dead space fraction

Standard formulae were used to calculate lung compliance. The alveolar dead space fraction was measured with the Cosmo Plus Respironics monitor (Novametrix Medical Systems, Wallingford, CT, USA) [21].

### Bronchoalveolar lavage

The bronchoscope was wedged in the distal airway. The initial 25 ml of warm saline injected was discarded. Five further 25 ml aliquots were instilled and aspirated. A portion of the aspirated fluid was spun at 1,500 × *g *for 10 minutes at 4°C. The supernatant was stored at -80°C. Samples were assayed by ELISAs for PTF levels (Enzygnost F1 + 2 monoclonal assays; Behring, Marburg, Germany) and for t-PA antigen levels (TintElize tPA, Biopool International, Ventura, CA, USA).

### Statistical analysis

Based on previous studies we determined that four patients in each group would be adequate to detect a major anticoagulant effect [23,24]. Analysis of variance was used to compare the effect of heparin dose on the P_a_O_2_/F_i_O_2 _ratio, lung compliance, the alveolar dead space fraction, the APTT, the TCT and intrapulmonary PTF and t-PA levels. Fisher's exact test compared categorical variables. Student's *t *test compared normally distributed variables.

Data are reported as the mean ± standard deviation. Statistical analysis was performed with the JMP program (SAS Institute, Inc., Cary, NC, USA).

## Results

### Patient characteristics

Sixteen patients were enrolled. The mean patient age was 58 ± 14 years, and the Acute Physiology and Chronic Health Evaluation Score II was 21 ± 7. The baseline P_a_O_2_/F_i_O_2 _ratio was 183 ± 66 mmHg, lung compliance was 26 ± 14 ml/cmH_2_O and the alveolar dead space fraction was 0.23 ± 0.1. Prophylactic subcutaneous heparin was administered to 14 of the 16 patients studied. The commonest aetiological factor for ALI was pneumonia (Table 1). The time from intubation to initial heparin nebulisation was 22 ± 15 hours. The mean mechanical ventilation time was 10 ± 9 days, the intensive care length of stay was 12 ± 8 days and the hospital length of stay was 28 ± 14 days. The tracheostomy rate was 63% and the hospital mortality was 43%.

**Table 1:** Baseline and microbiological characteristics of patients

Variable	Dose				Combined *n *(%)
	
	50,000 U/day	100,000 U/day	200,000 U/day	400,000 U/day	
Number of patients	4	4	4	4	16
Age (years)	57.7 ± 17.3	61.3 ± 14.2	54.3 ± 17.6	60.0 ± 13.0	58.3 ± 14.3
Male	2	1	4	1	8 (50)
Acute Physiological and Chronic Health Evaluation II score	22.3 ± 6.0	21.8 ± 8.7	18.3 ± 10	20.0 ± 3.4	20.6 ± 7.0
Subcutaneous heparin	4	3	4	3	14 (88)
Arterial to inspired oxygen ratio (mmHg)	159 ± 37	143 ± 48	207 ± 79	226 ± 75	183 ± 66
Lung compliance (ml/cmH_2_O)	30 ± 7	19 ± 5	37 ± 21	22 ± 14	26 ± 14
Alveolar dead space fraction	0.15 ± 0.04	0.3 ± 0.10	0.17 ± 0.07	0.24 ± 0.1	0.23 ± 0.1
Creatinine (μmol/l)	80 ± 45	56 ± 14	130 ± 154	97 ± 45	91 ± 80
Activated partial thromboplastin time (s)	38 ± 7	37 ± 6	44 ± 7	47 ± 20	41 ± 11
Thrombin clotting time (s)	17 ± 2	21 ± 3	20 ± 10	19 ± 3	19 ± 5
Acute lung injury cause					
Pneumonia	3	3	2	2	10 (63)
Sepsis	0	0	1	1	2 (13)
Pancreatitis	0	1	0	1	2 (13)
Empyema	1	0	1	0	2 (13)
Aspiration	0	0	0	0	0
Surgical admission	3	0	1	2	6 (38)
Microbiology					
Gram-negative	1	0	2	1	4 (25)
Gram-positive	1	2	1	0	4 (25)
Other	0	1	0	2	3 (19)
No pathogen detected	2	1	1	1	5 (31)
Blood culture-positive	0	0	1	1	2 (12)

### Lung function

**Figure 1:**
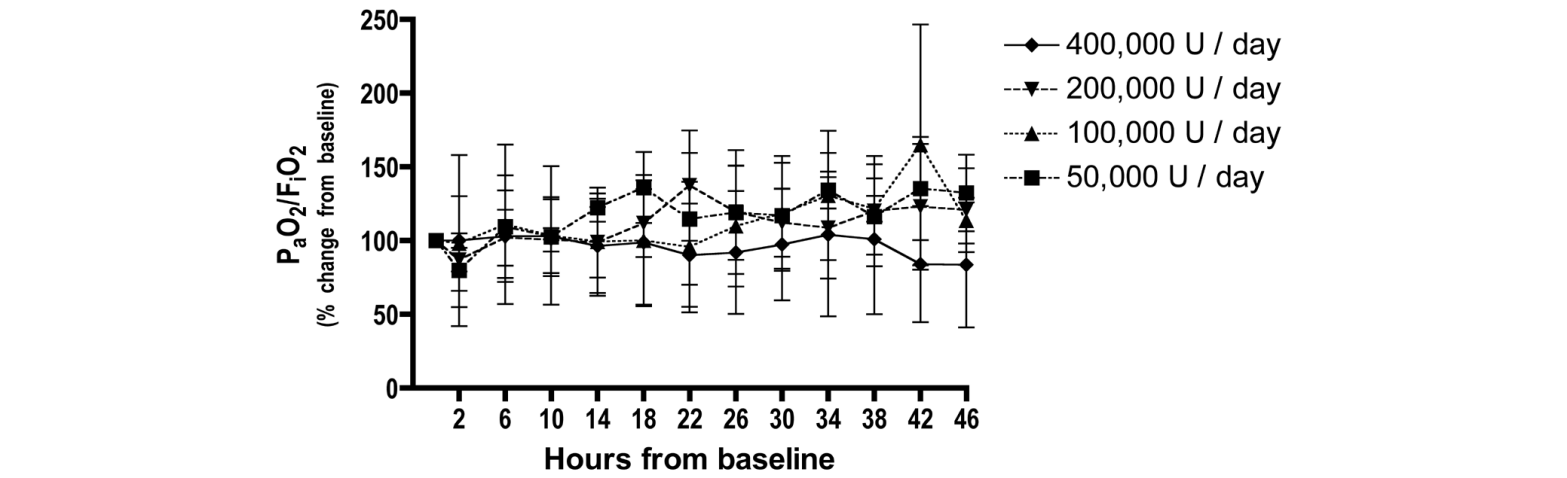
Changes in arterial to inspired oxygen ratio with nebulised heparin dosage. Percentage change from baseline in the arterial to inspired oxygen ratio (P_a_O_2_/F_i_O_2_) (mean ± standard deviation).

**Figure 2:**
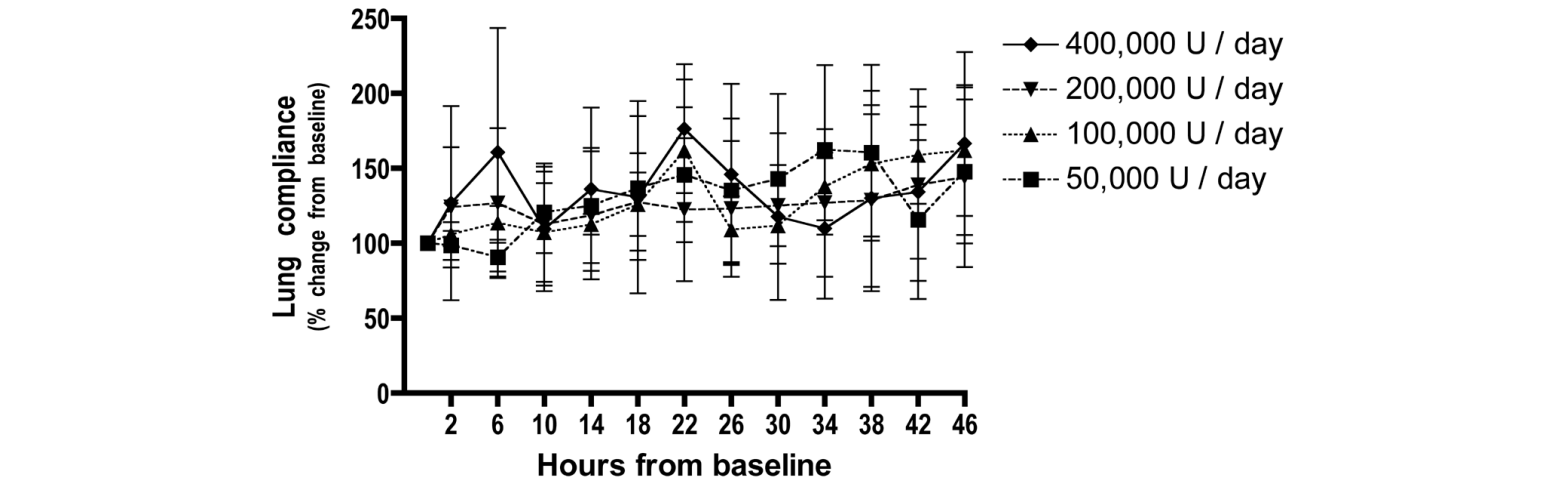
Changes in lung compliance with nebulised heparin dosage. Percentage change from baseline in the lung compliance over time for each dose (mean ± standard deviation).

**Figure 3:**
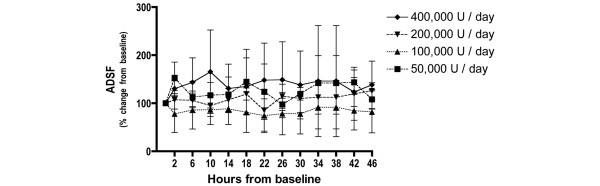
Changes in alveolar dead space fraction with nebulised heparin dosage. Percentage change from baseline in the alveolar dead space fraction (ADSF) (mean ± standard deviation).

The changes over time in the P_a_O_2_/F_i_O_2 _ratio, lung compliance and the alveolar dead space fraction were similar for all doses studied. There were no statistically significant differences found for the dosage or for the interaction between dosage and time (Figures 1 to 3).

One patient in the 400,000 U/day group developed blood-stained respiratory secretions after the seventh dose. This was not associated with any deterioration in lung function. The blood staining resolved following withdrawal of nebulised heparin.

### Anticoagulant effects

**Figure 4:**
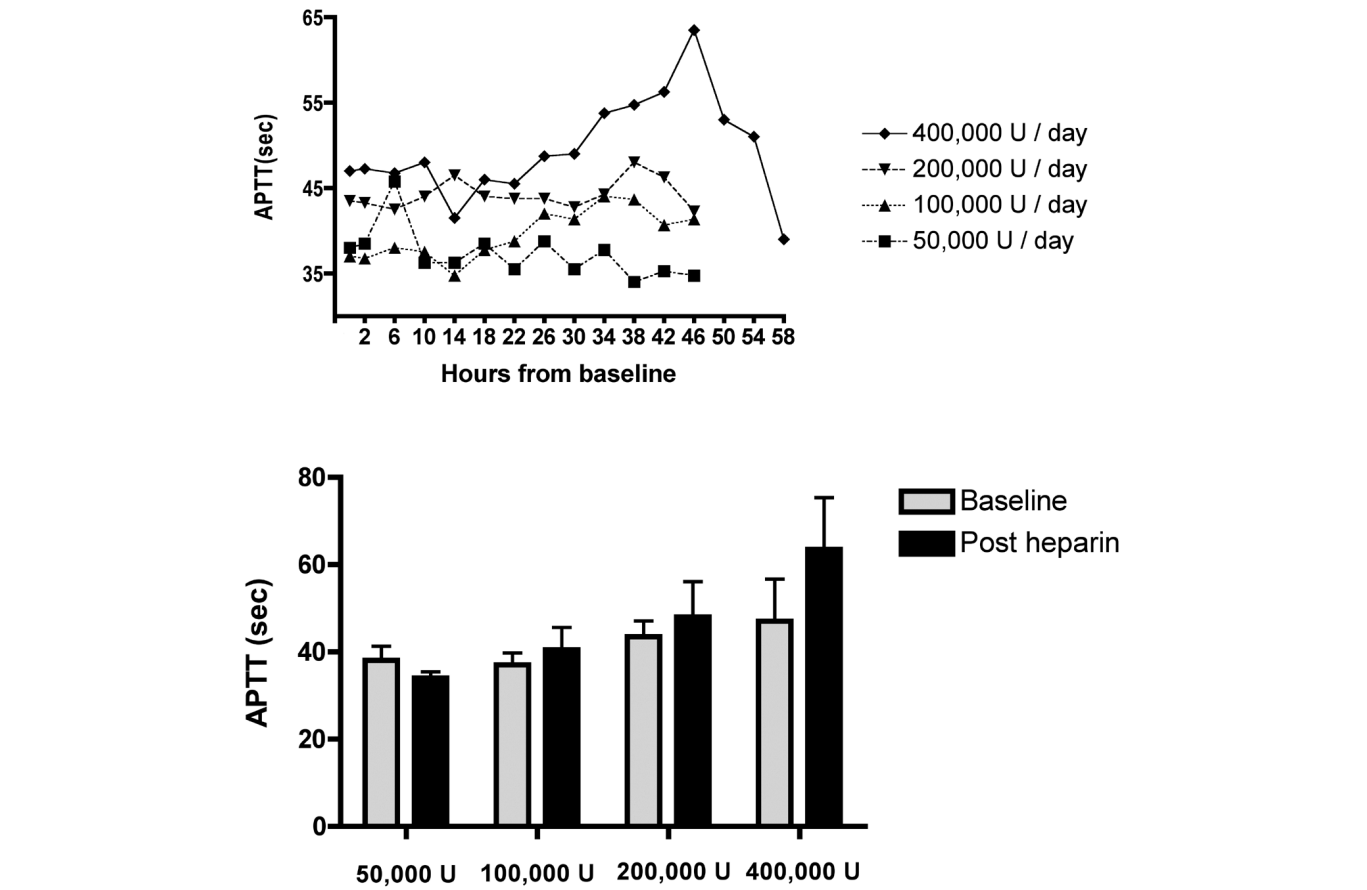
Changes in activated partial thromboplastin time with nebulised heparin dosage. Upper: Change in the activated partial thromboplastin time (APTT) over time. The last dose of heparin was given at 36 hours for all groups except the 400,000 U/day group, in which it was administered at 42 hours (mean levels; *P *= 0.06, comparison of 46-hour and 58-hour timepoints in the 400,000 U/day group). Lower: APTT at baseline and following the final dose of nebulised heparin (*P *= 0.09, analysis of variance comparison by dose, mean and standard deviation).

**Figure 5:**
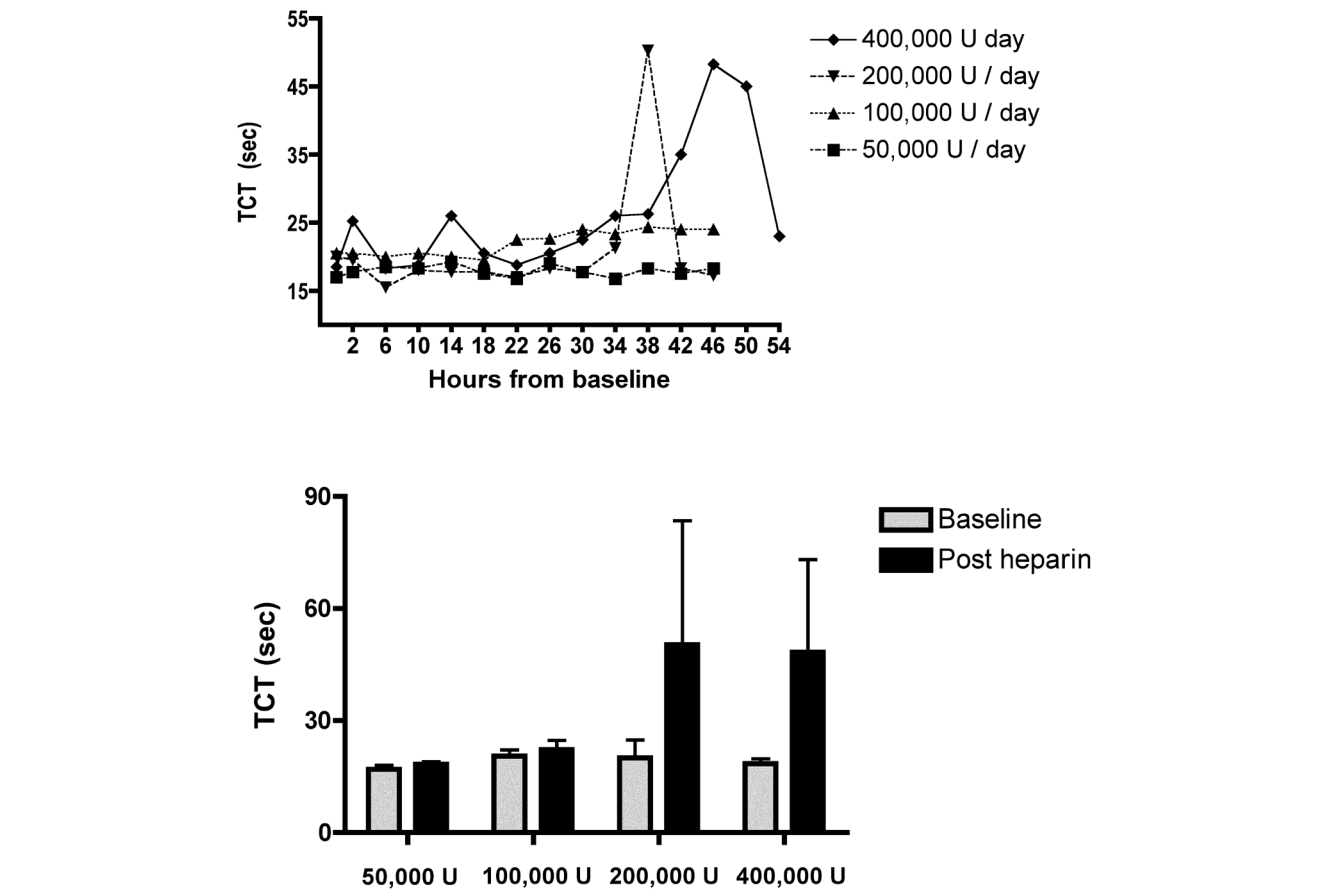
Changes in thrombin clotting time with nebulised heparin dosage. Upper: Change in the thrombin clotting time (TCT) over time. The last dose of heparin was given at 36 hours for all groups except the 400,000 U/day group, in which it was administered at 42 hours (mean levels). Lower: TCT at baseline and following the final dose of nebulised heparin (*P *= 0.1, analysis of variance comparison by dose, mean and standard deviation).

The mean APTT for each group following the final nebulisation, in order of increasing dose, was 34 seconds (normal range < 35 seconds), 41 seconds, 48 seconds and 64 seconds (*P *= 0.09, analysis of variance, comparison by dose) (Figure 4). The mean TCT for each group following the final nebulisation, in order of increasing dose, was 18 seconds (normal range < 21 seconds), 23 seconds, 50 seconds and 48 seconds (*P *= 0.1, analysis of variance, comparison by dose) (Figure 5). For the higher dose groups, both the APTT and TCT fell following cessation of nebulised heparin. For the highest dose, the APTT fell from 64 seconds to 39 seconds (*P *= 0.06) (Figure 4).

### Bronchoalveolar lavage

**Figure 6:**
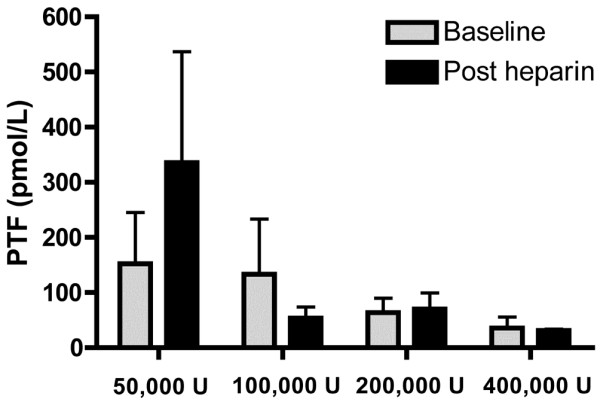
Comparison of prothrombin fragment levels in bronchoalveolar fluid with nebulised heparin dosage. Comparison of prothrombin fragment (PTF) levels in bronchoalveolar fluid. Levels were assessed at baseline and following the final dose of nebulised heparin (*P *= 0.1, analysis of variance comparison by dose, mean and standard deviation).

**Figure 7:**
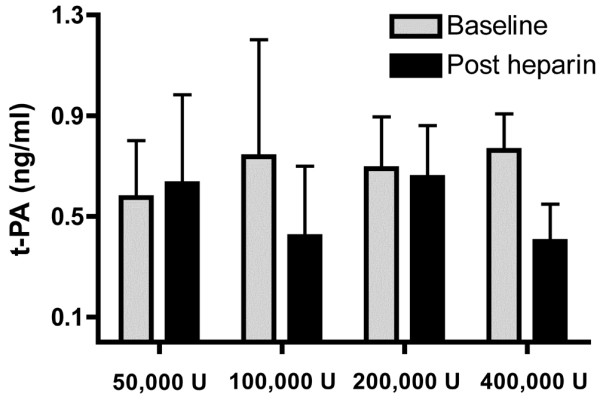
Comparison of tissue plasminogen activator levels in bronchoalveloar fluid with nebulised heparin dosage. Comparison of tissue plasminogen activator (t-PA) levels in bronchoalveloar fluid. Levels were assessed at baseline and following the final dose of nebulised heparin (mean and standard deviation).

The PTF levels in BAL fluid in the 50,000 U/day group were higher following the final nebulisation, while in the 100,000 U/day, 200,000 U/day and 400,000 U/day groups the PTF levels remained similar to baseline levels following the final nebulisation (*P *= 0.1, analysis of variance, comparison by dose) (Figure 6). The t-PA levels were similar to baseline levels for all doses following the final nebulisation (Figure 7).

## Discussion

We assessed the feasibility, safety and potential anticoagulant effects of nebulised heparin in mechanically ventilated patients with ALI. We found administration of nebulised heparin to mechanically ventilated patients with ALI was feasible, was not associated with serious adverse events, and increased APTT levels at higher doses.

The changes in the PaO_2_/FiO_2 _ratio, lung compliance and the alveolar dead space fraction were similar for all doses. In one patient in the 400,000 U/day group, blood staining of the respiratory secretions was present after the seventh dose. This staining resolved following withdrawal of heparin.

We found evidence of dose-dependent effects on APTT and TCT levels. For the 50,000 U/day group, the levels remained within the normal range; however, for the 100,000 U/day, 200,000 U/day and 400,000 U/day groups, the APTT and TCT levels were raised on the second day. Peak levels were reached following the final nebulisation, and thereafter levels fell. For the 400,000 U/day group, the APTT reached the therapeutic range (64 seconds) and fell acutely to 39 seconds following cessation of nebulised heparin.

Previous clinical studies have investigated the potential of systemic anticoagulation using nebulised heparin – to date, without success [23,24]. Unlike these studies we used repeated doses of nebulised heparin. Our finding that the APTT and TCT levels increased only after repeated doses suggests that pulmonary processes, such as storage of heparin in endothelial cells and metabolism by heparinases, may initially limit heparin reaching the systemic circulation. These processes may, however, become saturated following repeated heparin doses [25]. In future trials of nebulised heparin, due consideration must be given to this systemic anticoagulant effect.

We also examined whether nebulised heparin limited coagulation and increased fibrinolysis in the lungs. For the 50,000 U/day group, the PTF levels in BAL fluid doubled from baseline levels following the final nebulisation. For the 100,000 U/day, 200,000 U/day and 400,000 U/day groups, however, the PTF levels did not increase. Previous trials in patients with ventilated-associated pneumonia also found a doubling of coagulation levels in BAL fluid over the first few days [26,27]. While inconclusive, our findings raise the possibility that nebulised heparin, at higher doses, limited coagulation activation in the lungs. Nebulised heparin did not increase t-PA levels in BAL fluid for any of the doses studied.

One of the strengths of the present study was the nebulisation system used. The evidence of a dose-related effect on systemic APTT and TCT levels suggested significant amounts of heparin reached the alveolar spaces. This finding is consistent with previous studies [28]. Another strength of the study was the inclusion of genuinely high-risk patients with ALI. The average P_a_O_2_/F_i_O_2 _ratio at baseline was 183 mmHg, the tracheostomy rate was 63% and the hospital mortality was 43%.

Limitations of the present study included the absence of a control group, the small number of patients enrolled and the relatively short time (2 days) over which heparin was nebulised. The size of the study reflected the need for caution, as nebulised heparin had not previously been administered to patients with ALI. Furthermore, we determined that four patients in each group would provide adequate power to detect a major anticoagulant effect. Our study was consequently too small to draw conclusions regarding efficacy or potential infrequent deleterious effects.

Previous studies in animal models of ALI have demonstrated significant improvements in pulmonary function with inhaled heparin and other glycosaminoglycans [19,20,29]. Heparin has a range of anticoagulant actions and also promotes fibrinolysis through increased t-PA levels [15-18]. Compared with the intravenous route, nebulisation delivers high concentrations of heparin to the alveolar space with a reduced risk of adversely effecting systemic coagulation.

## Conclusion

Administration of nebulised heparin to mechanically ventilated patients with ALI is feasible. The heparin administration was not associated with any serious adverse events, and increased APTT levels at higher doses. Larger trials are required to further investigate the safety and efficacy of nebulised heparin in ALI. In these trials, due consideration must be given to systemic anticoagulant effects.

## Key messages

• Administration of nebulised heparin to mechanically ventilated patients with ALI is feasible and was not associated with any serious adverse events.

• At higher doses, nebulised heparin increased APTT levels.

• Larger trials are required to further investigate the safety and efficacy of nebulised heparin in ALI.

## Abbreviations

ALI = acute lung injury; APTT = activated partial thromboplastin time; BAL = bronchoalveolar lavage; ELISA = enzyme-linked immunosorbent assay; PaO_2_/FiO_2 _= arterial oxygen partial pressure to inspired oxygen fraction ratio; PTF = prothrombin fragments; TCT = thrombin clotting time; t-PA = tissue plasminogen activator.

## Competing interests

The authors declare that they have no competing interests.

## Authors' contributions

BD designed the study, collected the data, performed the statistical analysis and drafted the manuscript. JDS and DJC participated in its design, and coordinated and helped to draft the manuscript. All authors read and approved the final manuscript.
